# Species’ functional traits and interactions drive nitrate-mediated sulfur-oxidizing community structure and functioning

**DOI:** 10.1128/mbio.01567-23

**Published:** 2023-09-13

**Authors:** Tongchu Deng, Zhili He, Meiying Xu, Meijun Dong, Jun Guo, Guoping Sun, Haobin Huang

**Affiliations:** 1 State Key Laboratory of Applied Microbiology Southern China, Guangdong Provincial Key Laboratory of Microbial Culture Collection and Application, Institute of Microbiology, Guangdong Academy of Science, Guangzhou, China; 2 Guangdong Provincial Key Laboratory of Environmental Protection Microbiology and Regional Ecological Security, Guangzhou, Guangdong, China; 3 Southern Marine Science and Engineering Guangdong Laboratory (Zhuhai), Zhuhai, China; University of Massachusetts Amherst, Amherst, Massachusetts, USA

**Keywords:** species’ functional traits, microbial interactions, nitrate-mediated sulfur oxidation, community assembly, community function

## Abstract

Understanding processes and mechanisms governing microbial community structure and function is a central goal in microbial ecology. Previous studies disentangling the community assembly mechanisms were mainly based on taxonomic diversity but were rarely combined with species’ functional traits and interactions. Here, we showed how species’ functional traits and interactions determined microbial community structure and functions by a well-controlled laboratory experiment with nitrate-mediated sulfur oxidation systems using both culture-independent and culture-dependent technologies. The results showed that species were different in functional traits of nitrate-mediated sulfide and thiosulfate oxidation, which determined their relative abundance in the nitrate-mediated sulfur oxidation systems. Those thiosulfate-oxidizing microbes co-occurred with *Thiobacillus* by using intermediates (e.g., thiosulfate) secreted by *Thiobacillus* during sulfide oxidation process. Such metabolic dependencies exerted great effects on community functions. Metabolic dependencies between *Thiobacillus* and genera that oxidized thiosulfate to more sulfate (e.g., *Ciceribacter*) sustained high and stable oxidation activities of sulfide to sulfate. In contrast, metabolic dependencies between *Thiobacillus* and genera that oxidized thiosulfate to tetrathionate (e.g., *Pseudoxanthomonas*) slowed down the production of sulfate, indicating changes in the metabolic flow. In addition, competitions among species were mostly detrimental to the stability of community function. These results revealed that species’ functional traits and interactions were the intrinsic factors determining community structure and functions. This study advances our understanding of microbial community assembly and functions of the nitrate-mediated sulfur oxidation process from the perspectives of species’ functional traits and interactions and has important implications for designing and constructing microbiomes with expected functions.

**Importance:**

Understanding the processes and mechanisms governing microbial community assembly and their linkages to ecosystem functioning has long been a core issue in microbial ecology. An in-depth insight still requires combining with analyses of species’ functional traits and microbial interactions. Our study showed how species’ functional traits and interactions determined microbial community structure and functions by a well-controlled laboratory experiment with nitrate-mediated sulfur oxidation systems using high-throughput sequencing and culture-dependent technologies. The results provided solid evidences that species’ functional traits and interactions were the intrinsic factors determining community structure and function. More importantly, our study established quantitative links between community structure and function based on species’ functional traits and interactions, which would have important implications for the design and synthesis of microbiomes with expected functions.

## INTRODUCTION

Microbial communities play critical roles in matter circulation and energy transformation in natural ecosystems (1 – 3). Understanding the factors and mechanisms determining the diversity, structure, function, and interaction of microbial communities is of great importance for predicting microbial community dynamics and controlling microbial communities to better serve the ecosystem and human health (4 – 6). Therefore, discovering the factors and mechanisms governing microbial community assembly and their linkages to ecosystem functioning has long been a core issue in microbial ecology (7).

Applications of large-scale high-throughput metagenomics technologies have revealed that microbial communities are highly complex and dynamic in natural ecosystems, such as extremely high diversity patterns (8 – 10), spatiotemporal turnover of species composition (10), and co-occurrence of phylogenetically conserved species (11, 12). Different from macrobiota, it is difficult to determine the desired microbial traits due to their small sizes and the highly complex community they are inhabiting (12, 13). Existing studies mainly used the taxonomic or phylogenetic diversity of microbial communities for inferring the effects of ecological processes on the microbial community structure (13 – 18). However, such efforts may not be sufficient to explore mechanisms for the formation of diverse microbial communities caused by deterministic and stochastic factors. Therefore, a profound understanding of community assembly mechanisms still needs the integration of traits-based analyses, which has been well evidenced for the macrobiotic communities but is still lacking for microbial communities (12, 19 – 21). In addition, a microbial community is characterized by highly complex interspecies interactions (e.g., competition, facilitation, mutualism, etc. [22, 23]). As the direct determination of microbial interactions is challenging, current methods mainly infer by association analyses, which may be susceptible to environmental changes and network construction algorithms (22, 23). At present, mechanistic understanding of the microbial interactions determining community assembly is still lacking.

Beyond the community assembly, linking the community structure to ecosystem functioning is another core issue of microbial ecology (7). Many previous studies have revealed that species abundance, diversity, and interactions of microbial communities are closely related to ecosystem functions (10, 24 – 26). Nevertheless, due to the challenge of direct measurement of species’ functional traits in the microbial community, previous studies linked community structure to ecosystem function based mainly on association analyses of species relative abundances with environmental variables (27). However, random events (e.g., stochastic processes of species, the upheaval in environmental conditions) may disrupt these associations by affecting the metabolic processes, microbial interactions, and the turbulence of community structure. A clear and quantitative linkage between community structure and ecosystem function also needs the integration of direct analyses of species’ functional traits and their interactions (20, 21).

The nitrate-mediated sulfur oxidation is an autotrophic process widely distributed in natural habitats such as sediments, soils, hydrothermal vents, deep sea redox transition zones, inland soda lakes, etc. (28 – 32). This process coupled sulfur compounds oxidation with nitrate reduction, which can drive the detoxification of sulfide and the removal of nitrogen (32 – 35). Furthermore, the generation of sulfate from sulfur oxidation can promote the degradation of organic pollutants by sulfate reduction (36, 37). Therefore, the nitrate-mediated sulfur oxidation process plays a critical role in pollutant removal in contaminated environments (28, 33). Similar to most microbial communities, the communities inhabited by nitrate-mediated sulfur oxidizing bacteria are highly diverse and perform distinct functions (28 – 32). However, mechanisms shaping this autotrophic community structure and function remain rarely explored. Little information was available about the taxonomy and functional traits of nitrate-reducing, sulfur-oxidizing bacterial species and their linkages to the nitrate-mediated sulfur oxidation process. It was found that the habitats that prefer autotrophic microbes are usually resource-limited, and deterministic processes are considered to dominate community assembly in harsh conditions (38, 39). In other words, species’ functions and interactions may play a critical effect on structuring the nitrate-mediated sulfur oxidation communities.

The combination of well-controlled laboratory experiments (e.g., dilution culturing) and high-throughput sequencing technologies may overcome the difficulty in analyzing species’ functional traits and interactions in microbial communities (40, 41). In this study, we imitated the assembly of nitrate-mediated sulfur-oxidizing communities by series of dilution culturing systems and linked their structure to the overall sulfur oxidation process. We aimed to understand how species’ functional traits and interactions drive the structure and functioning of nitrate-mediated sulfur-oxidizing community. We hypothesized that (i) species involved in nitrate-mediated sulfur oxidation were different in their functional traits of sulfur compounds oxidation and nitrate reduction, which determined their occurrence in the community, and (ii) these functionally different species co-occurred via metabolic interdependence in the nitrate-mediated sulfur oxidation process, and their coexistence affected the overall sulfur oxidation process of the system. We systematically tested those hypotheses by combining culture-independent with culture-dependent techniques, including high-throughput sequencing, genomic analyses, pure culturing, and co-culturing. The results would advance our understanding of microbial community assembly in the nitrate-mediated sulfur oxidation process from the perspectives of species’ functional traits and interactions. Moreover, we established quantitative links between community structure and its function based on species’ functional traits and interactions, which have important implications for the rational design and synthesis of microbial communities for their application in fields from environmental protection to metabolic engineering (42).

## MATERIALS AND METHODS

### Sources and environment conditions of the regional species pool

The regional species pool was derived from the surface sediment of a river (22°45'31"N, 113°16'21"E) in the Pearl River Delta. The sediment was located at ~50 cm below the water surface, where the dissolved oxygen had dropped to zero. Sediment columns were sampled and kept in bottles with N_2_ as blanket gas. The coexistence of nitrate and acid-volatile sulfide suggested that the *in situ* sediment was a suitable habitat for nitrate-mediated sulfur-oxidizing microorganisms (Table S1A). The potential nitrate-mediated sulfur oxidation activities also supported the existence of such microorganisms in the sediment environment (Text S1A; Table S1A).

### Imitation of community assembly by dilution culturing

Nitrate-mediated sulfide-oxidizing and thiosulfate-oxidizing communities were assembled by dilution culturing (Fig. S1) using media containing sulfide (NS medium) and thiosulfate (NT medium) as sole electron donor, respectively. NS medium contains the following ingredients per liter of deionized water: 2 g KNO_3_, 1 g Na_2_S·9H_2_O, 1 g NaHCO_3_, 0.5 g FeCl_2_, 2 g KH_2_PO_4_, and 0.1 g MgCl_2_. NT medium contains 2 g KNO_3_, 5 g Na_2_S_2_O_3_·5H_2_O, 1 g NaHCO_3_, 0.1 g FeCl_2_, 2 g KH_2_PO_4_, and 0.1 g MgCl_2_ per liter of deionized water. Na_2_S·9H_2_O, NaHCO_3_, FeCl_2_, and MgCl_2_ were added into the medium after filtering each mother solution through 0.22 µm membrane, respectively. The medium pH was adjusted to 7.0. Then 1 g of sediment was added to 10 mL of medium in a 20 mL headspace bottle to create the 10^−1^ dilution, and the other seven serial dilutions were made by adding 1 mL sample of the previous dilution to 9 mL of medium. These diluted samples represented the result of dispersal limitation caused by the different abundance of species. A total of 12 replicates were set for evaluating the randomness. After blowing by N_2_ to remove oxygen, all samples were placed in an anaerobic chamber and stationary cultured at 30°C. The concentrations of nitrate, thiosulfate, and sulfate were measured every 5 days to monitor functional activities. When the concentrations of these substrates did not change significantly, all bacterial cells were transferred to fresh media and cultured twice to achieve stable communities. Finally, an equal biomass of microbes from each sample, adjusted by estimating the number of bacterial cells under light microscope using hemocytometers (43), was transferred to fresh media to compare their functional activities by evaluating the consumption of nitrate and the generation of sulfate (Text S1B).

### Community structure analyses by 16S rRNA gene amplicon sequencing

The dilution-defined communities that showed different activities in nitrate-mediated sulfur oxidation were selected for community structure analyses. Original sediment and several communities without obvious functional activity were also chosen for comparison. Viable bacteria DNA was extracted and purified by the protocol described previously (44) after being treated with propidium monoazide to remove dead cell DNA (45). The V4 region of the 16S rRNA gene was amplified by barcoded primers (515F and 806R) using the protocol described previously (46). The amplicons were separated by 1% agarose gel electrophoresis and extracted from gels using a gel extraction kit (TransGen Biotech Co., Ltd, China). All products were pooled, cleaned, and normalized by VAHTS Nano DNA Library Prep Kit (Vazyme Biotech Co., Ltd, China) and sequenced on an Illumina MiSeq. Raw reads were demultiplexed and proceeded with bioinformatics analyses using QIIME 2 (47) with the Silva 16S rRNA gene database. Amplicon sequence variants (ASVs) were generated with 100% similarity. All samples were rarefied to 5,000 reads per sample to normalize the sequencing depth.

### Estimating influences of ecological processes on community structure

We used a null modeling-based statistical framework developed by Stegen et al. (17) to quantify the contribution of various ecological processes in structuring microbial community in our systems. The phylogenetic and taxonomic turnovers were measured with null model-based phylogenetic β-diversity metrics (βNTI) and taxonomic β-diversity metrics (RC_bray_) for all pairwise community comparisons. First, βNTI values less than −2 indicated significantly less than expected phylogenetic turnover between pairwise communities due to homogeneous selection. In contrast, βNTI values greater than 2 indicated significantly greater than expected phylogenetic turnover between pairwise communities resulting from the heterogeneous selection. Pairwise comparisons with |βNTI| less than 2 were assigned to stochasticity (e.g., dispersal limitation, homogenizing dispersal, and drift), which were subsequently partitioned using the taxonomic β-diversity metric RC_bray_. Dispersal limitation led to greater than expected turnover with |βNTI| less than 2 and RC_bray_ value of greater than 0.95, whereas homogenizing dispersal led to less than expected turnover with |βNTI| less than 2 and RC_bray_ value of less than −0.95. Pairwise comparisons with |βNTI| less than 2 and |RC_bray_| less than 0.95 were treated as “undominated” fractions, mainly drift acting alone.

### Species’ functional traits analyses by pure culturing and genome sequencing

The representative strains of predominant species were isolated by 1.5% agar plates using NS and NT media. The 16S rRNA gene of each strain was amplified and sequenced by universal primers 27f and 1492r using a protocol described by Frank et al. (48). Corresponding sequences were searched against the National Center for Biotechnology Information online database using Basic Local Alignment Search Tool for taxonomic identification. All strains were tested for functional traits, including nitrate-mediated sulfide, sulfur, and thiosulfate oxidations (Text S1C).

The genomes of all isolated strains were respectively extracted using an EasyPure Genomic DNA Kit (TransGen Biotech Co., Ltd, China) and sequenced on the Illumina HiSeq platform. The resulting reads were assembled using SPAdes v3.10.1 (49). Open reading frames were identified by Prodigal v3.13.1 (50). Coding sequences were annotated with eggNOG-mapper and BlastKOALA online tools (51, 52) for screening genes involved in the nitrate-mediated oxidation of reduced inorganic sulfur.

### Analyses of interspecies interactions by co-culturing

Different microbes co-occurred in a community suggesting the sign of metabolic interactions between these taxa (22, 24). The interspecies’ interactions on the nitrate-mediated sulfide oxidation process between different predominant ASVs were testified by two strains co-culturing experiment in comparison with each mono-culture. After culturing on agar plates containing NT medium, all involved strains were individually collected and inoculated into 10 mL NS liquid medium with the same level of biomass for each strain. After blowing by N_2_ to remove oxygen, all samples were placed in an anaerobic chamber and stationary cultured at 30°C. The metabolic activity of each treatment was characterized by measuring the production of sulfate at 24 and 96 h. Bacterial growth was monitored by quantitative polymerase chain reaction of partial 16S rRNA gene using the specific primers set for each strain (Text S1D; Table S1B).

### Estimation of community function by the summation of abundance-weighted species function

To analyze the contribution of species function to community function, we briefly estimated the community’s functional activities by the summation of species’ functional activities using the following formula (equation 1),


(equation 1) $$F\left(e\right)=\;\sum_{x=i}^{n}f\left(xi\right)a\left(xi\right)$$

in which *F*(*e*) was the estimated community’s functional activities, *f*(*xi*) represented species’ functional activities, and *a*(*xi*) represented the percentage abundance of species in community. Linear regression analyses accompanied by coefficient tests were employed to test the consistency of the estimated and the observed community functional activities.

### Statistical analysis

Shannon’s index was calculated to represent the alpha-diversity of each community using the ASV table. Community dissimilarities between groups were analyzed by the principal co-ordinates analysis (PCoA) based on the Jaccard distance and tested by the permutational multivariate analysis of variance (PERMANOVA). Alpha-diversity analyses, PCoA, and PERMANOVA were performed in R using the vegan package (53). Significance between groups was calculated by one-way ANOVA or independent samples *t*-test.

## RESULTS

### Variation of community structures and functions along a dilution gradient

By performing dilution culturing with two types of media, we successfully obtained two groups (sulfide oxidation and thiosulfate oxidation) of dilution culturing communities, respectively, with sulfide- and thiosulfate-oxidizing abilities under nitrate-reducing condition (Fig. S2). Significant (*P* < 0.05) differences in communities’ functional activities were observed along the dilution gradient (Fig. S2). Moreover, large variances were observed between replicates of more diluted samples, especially in the thiosulfate oxidation group (Table S1C). These variances indicated large differences in microbial community structures of more diluted samples.

**Fig 1:**
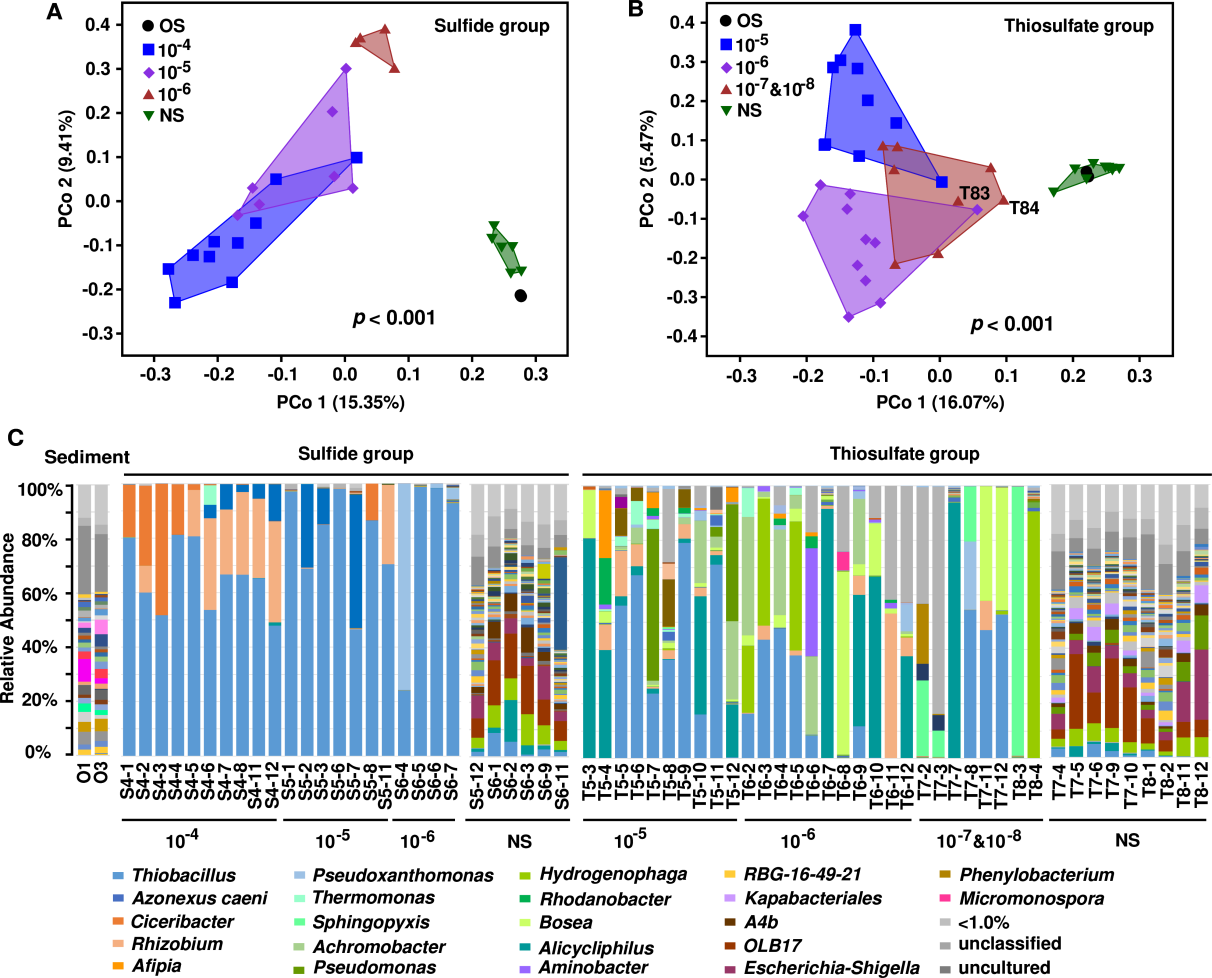
Divergent composition of dilution culturing communities. Community dissimilarity analyses based on Jaccard index to show divergent distribution pattern between different dilutions and among every dilution in both sulfide (**A**) and thiosulfate groups (**B**). Genus composition for communities of sulfide group and thiosulfate group (**C**). OS, original sediment; NS, communities with no significant activity.

We then analyzed how these communities differed in the structure by 16S rRNA gene amplicon sequencing. Compared to the original sediments and the communities without obvious activities of nitrate-mediated sulfur oxidation, the dilution communities with obvious sulfide or thiosulfate oxidation activities had significantly lower Shannon’s diversity indices (*P* < 0.001, Fig. S3). We further focused on those dilution culturing communities with desirable functional activities. Consistent with the variance of community function, the structure of dilution culturing communities was significantly (*P* < 0.001) divergent along dilution gradients in both groups (Fig. 1A and B). Moreover, divergences in composition were also observed between communities that belonged to the same dilution (Fig. 1A and B). *Thiobacillus* was a predominant genus in nearly all sulfide oxidation communities. Other predominant genera included *Ciceribacter*, *Rhizobium*, *Azonexus*, and *Pseudoxanthomonas* along dilution gradients, and one or two of these genera coexisted with *Thiobacillus* in a community, accounting for >94% relative abundance (Fig. 1C).

The microbial communities of the thiosulfate oxidation group were more complex than the sulfide oxidation group. There were many predominant genera in each thiosulfate oxidation community (Fig. 1C). Most genera, including *Thiobacillus*, *Alicycliphilus*, *Pseudoxanthomonas*, *Thermomonas*, *Hydrogenophaga*, *Rhodanobacter*, *Bosea*, and *Rhizobium,* were predominant in multiple dilution communities. In contrast, other genera were predominant only in specific dilution communities (e.g., *Afipia*, *Pseudomonas*, and *Denitratisoma* in 10^−5^; *Achromobacter* in 10^−6^; *Sphingopyxis* in 10^−7^ and 10^−8^ dilutions). Different assemblies constituted by the above genera were diverse not only between different dilutions but also within the same dilution. The community compositions of both sulfide and thiosulfate oxidation groups were also diverse at the family level (Fig. S4) and ultimately converged at the class level (Fig. S5).

### Selection dominated the formation of dilution culturing communities

**Fig 2:**
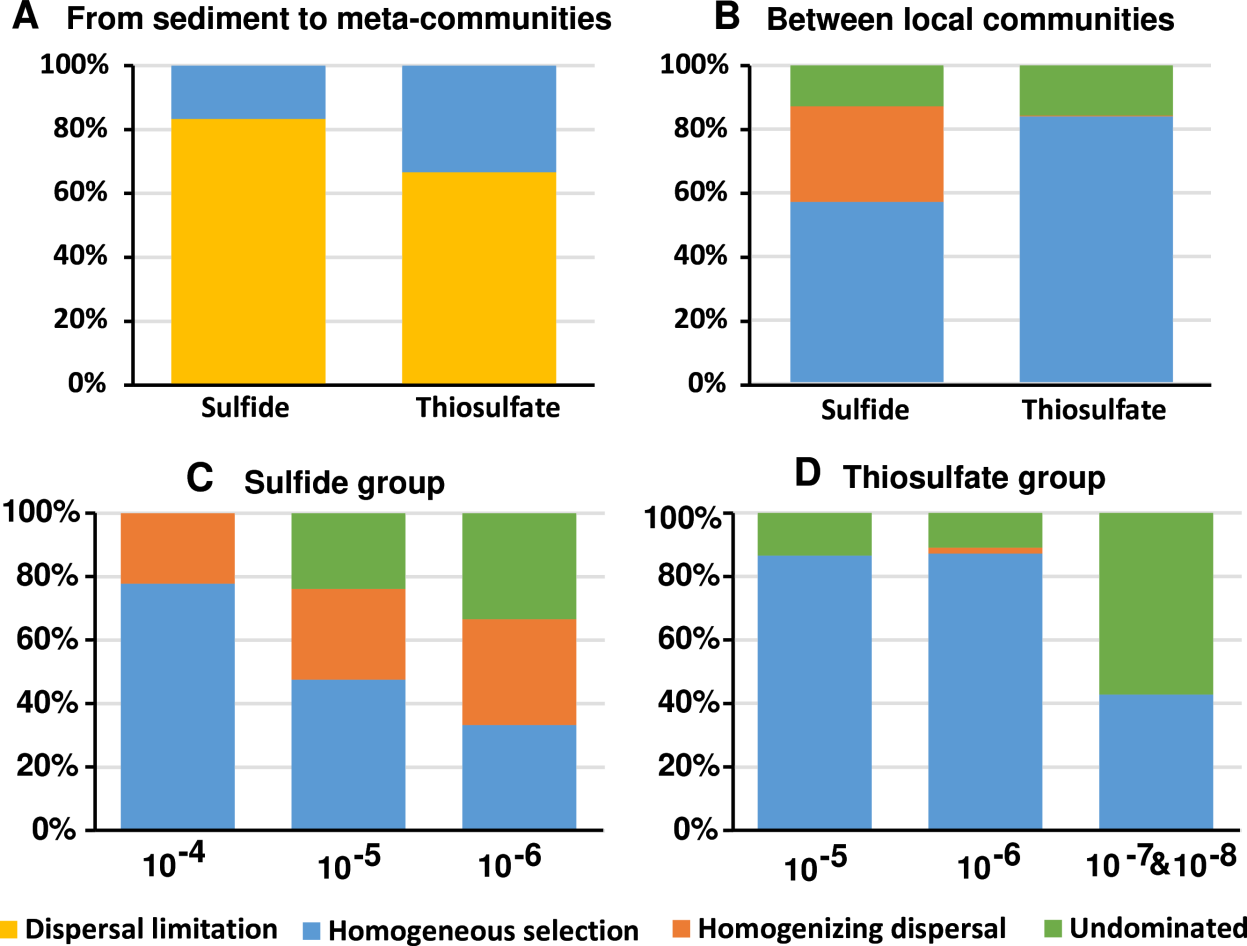
Ecological processes shaped the community structure of the sulfide and thiosulfate groups. (**A**) Ecological processes shaped the initial colonization of species in the dilution communities; (**B**) ecological processes led to divergences between communities with functional activity. Ecological processes shaping community structure of different dilutions for sulfide (**C**) and thiosulfate groups (**D**).

Since all communities were incubated under identical conditions, the only variables resulting in divergent community structure were deterministic factors such as species’ function and their interactions, as well as the stochastic process of dispersal caused by dilution. Therefore, we quantified the contribution of the ecological processes shaping community structure using the framework described by Stegen et al. (17). The effects of the dilution process on the community structure were characterized by analyzing the factors leading to the differences between species pool (sediment) and those communities without obvious nitrate-mediated sulfur oxidation activity (no obvious activity). The results showed that the differences were mainly caused by dispersal limitation (Fig. 2A), suggesting that dispersal exerted an effect on the initial colonization of species in the dilution communities.

Then, the processes governing the communities with the nitrate-mediated sulfur oxidation activity (with activity) were analyzed by pairwise comparisons. The results showed that homogeneous selection, homogenizing dispersal, and “undominated” fractions, respectively, accounted for 57%, 30%, and 13% of the community assembly within communities in the sulfide group. Those three processes accounted, respectively, for 84%, 0.2%, and 15.8% of the thiosulfate oxidation group (Fig. 2B). Notably, the contribution of homogeneous selection decreased, while homogenizing dispersal and “undominated” increased from low to high dilutions (Fig. 2C and D). These results suggested that selection played a major role in the later formation of divergent communities.

### Species’ functional traits determines their relative abundance in the communities

Ecological process analyses had revealed that selection was an important factor leading to the divergence of local communities. Because all communities were diluted from the same species pool and were incubated under identical environmental conditions, species’ functional traits were the inner factor for selection. We then analyzed how species’ functional traits determined nitrate-mediated sulfide and thiosulfate oxidation community structure by pure culturing and genome analyses.

**Fig 3:**
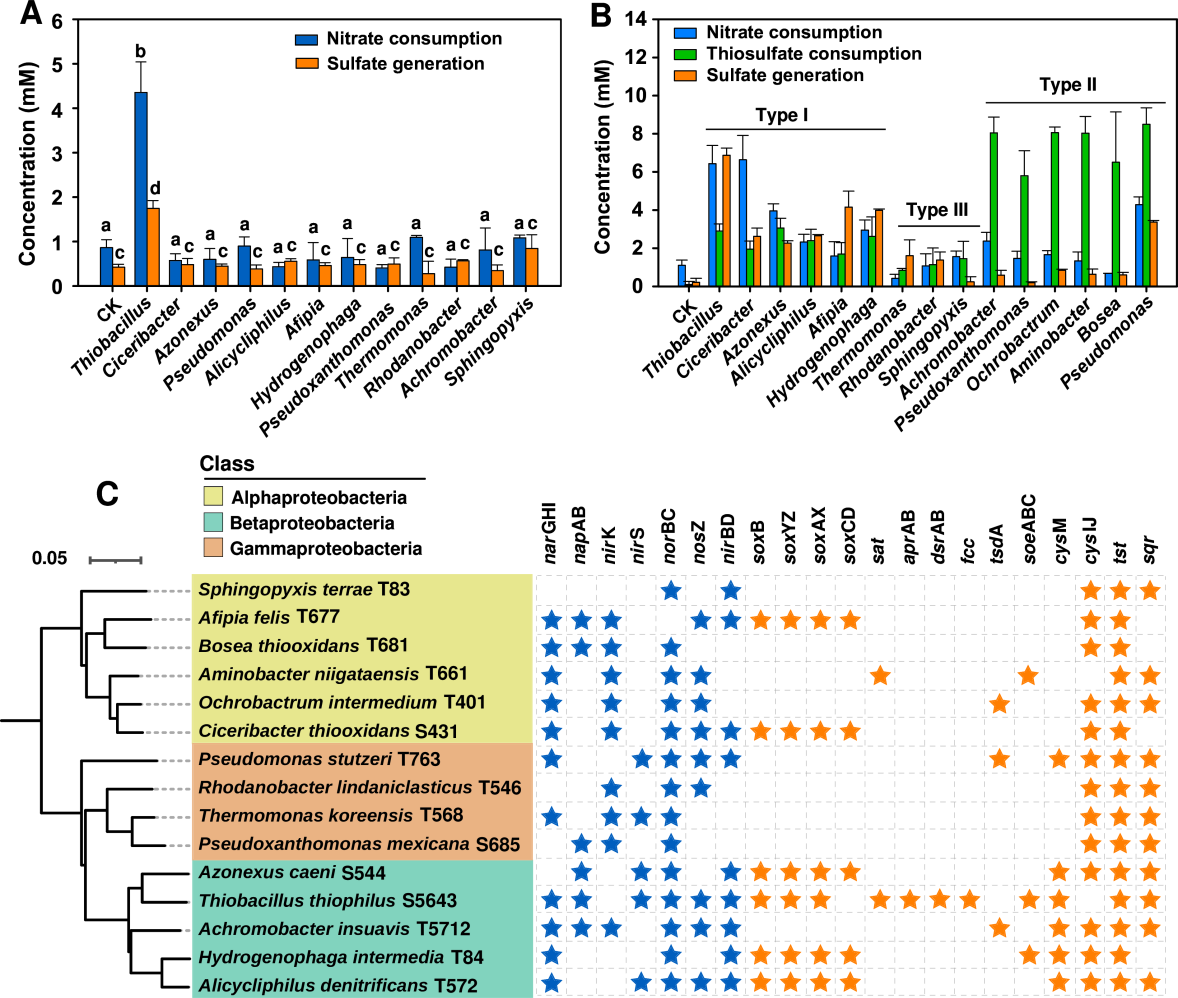
Functional traits, phylogenetic relationship, and functional genes involved in the nitrate-mediated sulfur oxidation of isolated strains. (**A**) Nitrate-mediated sulfide oxidation traits of the isolated strains after 4 days of incubation; a, b, c, and d represented significant differences between groups with *P* < 0.05 by independent samples *t*-test based on three samples for each group. (**B**) Nitrate-mediated thiosulfate oxidation traits of the isolated strains after 4 days of incubation. (**C**) 16S rRNA gene phylogenetic relationship and functional genes involved in nitrate-mediated sulfur oxidation for the isolated strains.

We successfully isolated 15 strains from the sulfide oxidation and thiosulfate oxidation dilution communities. The isolated strains represented 15 predominant genera in the communities and differed in the functional traits of nitrate-mediated sulfur oxidation (Fig. 3A and B). The strain that belonged to the genus *Thiobacillus* was the only one that had sulfide oxidation abilities under the nitrate-reducing condition (Fig. 3A). Genomic analyses revealed that this strain was the only one harboring *apr*AB, *dsr*AB, and *fcc* genes for sulfide oxidation (Fig. 3C), which explained why this genus was predominant in communities of the sulfide group (Fig. 1C). All the isolated strains had the thiosulfate oxidation ability (Fig. 3B), which probably explained why they presented in thiosulfate oxidation communities (Fig. 1C). These results showed different niches-selected species with adaptive functions.

According to the different functional traits of thiosulfate oxidation and nitrate reduction, all isolated strains could be divided into three types (Fig. 3B), including those oxidizing thiosulfate to more sulfate with much nitrate consumption (Type I), those oxidizing thiosulfate to tetrathionate and generating less sulfate (Type II, Fig. S6), and those oxidizing thiosulfate to less sulfate with only a little nitrate consumption (Type III). These functional differences in the isolated strains were evidenced by different genes involved in thiosulfate oxidation in their genomes (e.g., *sox* system, Fig. 3C). These functional traits of thiosulfate oxidation and nitrate reduction were closely linked to their relative abundance in the community. Type I genera were more frequently predominant in the communities of the thiosulfate group (Fig. 1C). In contrast, genera of Type III presented only in a few communities where Type I and Type II were absent (Fig. 1C), indicating that there may be competition among the different genera for dominance in the community.

### Metabolic interactions on sulfur oxidation lead to species coexistence

**Fig 4:**
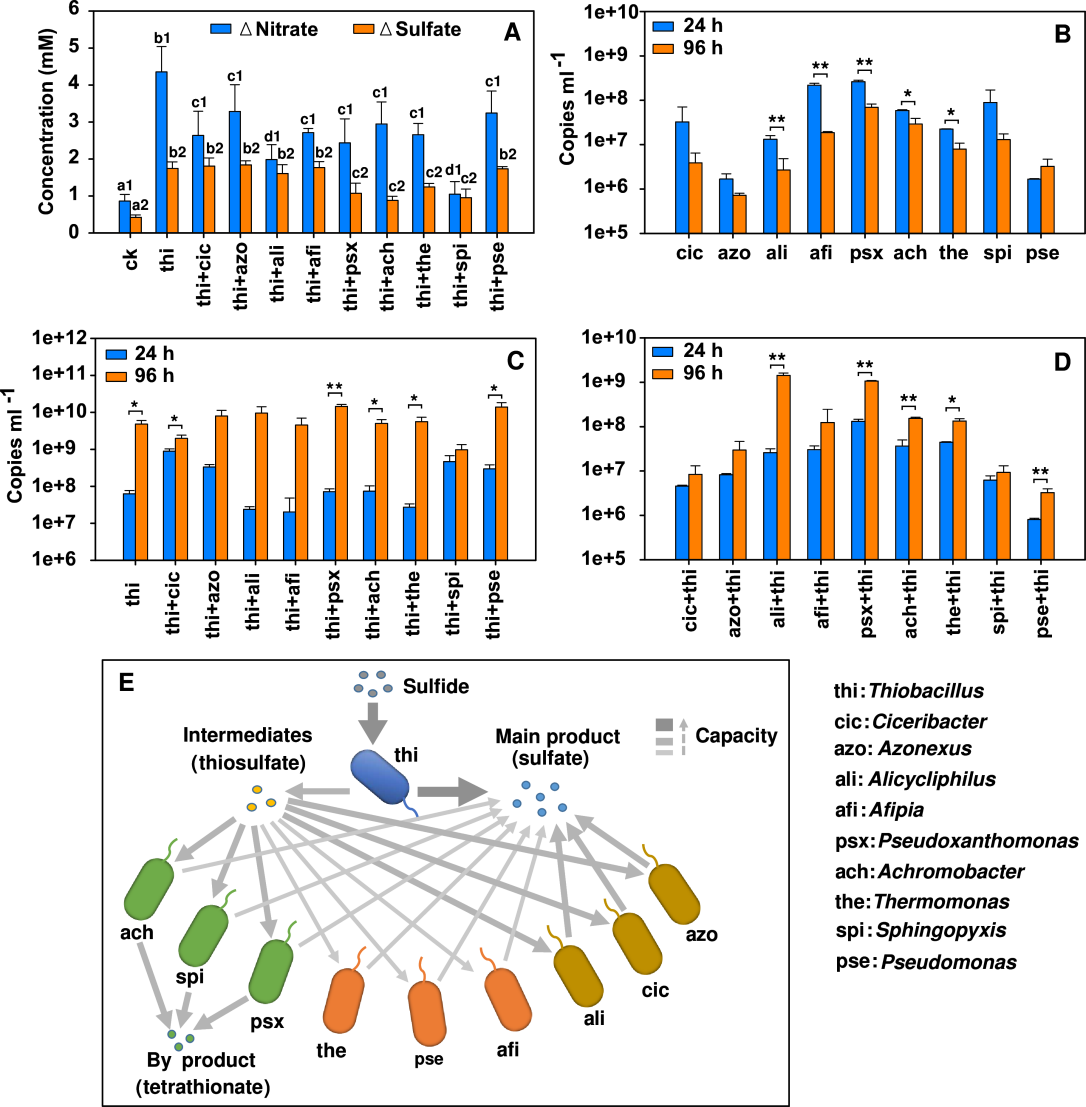
Co-culturing experiment showing metabolic dependencies of thiosulfate oxidation strains on the strain of *Thiobacillus*. (**A**) Nitrate consumption and sulfate production in co-culturing, mono-culturing, and abiotic control (ck) systems after 4 days of incubation; (**B**) decreased 16S rRNA gene copy numbers for strains in the mono-culture; (**C**) increased 16S rRNA gene copy numbers of *Thiobacillus*; (**D**) increased 16S rRNA gene copy numbers for strains that co-cultured with *Thiobacillus*; (**E**) schematic showing the metabolic dependencies of thiosulfate oxidation strains on *Thiobacillus* and the effects of metabolic dependencies on sulfide oxidation; a1, b1, c1, a2, b2, and c2 represented significant different groups with *P* < 0.05 by one-way ANOVA; *, *P* < 0.05, **, *P* < 0.01 by independent samples *t*-test. Significances were tested based on three samples for each group.

Some genera in the sulfide oxidation communities, such as *Ciceribacter* and *Azonexus*, could not utilize sulfide, and their occurrence could not be attributed to the sulfide-oxidizing function. We presumed that the sulfide oxidizer, *Thiobacillus*, might produce some metabolic intermediates to support the growth of these genera. For instance, thiosulfate had been proven to be a common metabolic intermediate of *Thiobacillus* (54). Consistently, all co-occurred genera showed thiosulfate oxidation ability and contained genes for thiosulfate oxidation (Fig. 3B and C), which supported their metabolic dependencies on *Thiobacillus*. The metabolic interactions between *Thiobacillus* and other thiosulfate-oxidizing genera were further confirmed by a series of two strains co-culturing experiments (Fig. 4; Fig. S7).

Solely culturing in NS medium, the 16S rRNA gene copy numbers of most thiosulfate-oxidizing genera decreased (Fig. 4B). In contrast, when they were co-cultured with *Thiobacillus*, their copy numbers increased (Fig. 4D), accompanied with the generation of sulfate (Fig. 4A) and the increase of the copy number of *Thiobacillus* (Fig. 4C). Moreover, we added iodine (I_2_) into the culture, with a final concentration of 10 mg/L, to remove thiosulfate after 2 days of culturing *Thiobacillus* in medium NS (Fig. S7B), and the resulting culture solution was filtered and used for culturing thiosulfate oxidizers (Text S1E). The results showed that those thiosulfate oxidizers grew worse than those cultures without iodine addition (Fig. S7C) and there was no obvious sulfate production (Fig. S7A). Notably, the worsened growth was not due to any inhibition effect of iodine because those thiosulfate oxidizers grew well when glucose was added to the filtrated culture solution (Fig. S7D). These results provide convincing evidences that thiosulfate was one of the intermediates produced by *Thiobacillus* which supported the growth of other thiosulfate oxidizers.

From the taxonomy perspective, the isolates with similar functional traits and genetic composition of nitrate-mediated sulfur oxidation were not conserved even at the class level. For instance, *Ciceribacter* and *Azonexus* belong to the classes Alphaproteobacteria and Betaproteobacteria, respectively, and they were both dominant in many sulfide oxidation communities. They both contain similar *sox* gene systems (Fig. 3C) which supported their metabolic dependencies on *Thiobacillus* (Fig. 1C). The taxonomy divergence of species with a similar function was the reason leading to divergent communities at higher taxonomy levels.

### Metabolic interactions lead to different metabolic fluxes of sulfide oxidation

**Fig 5:**
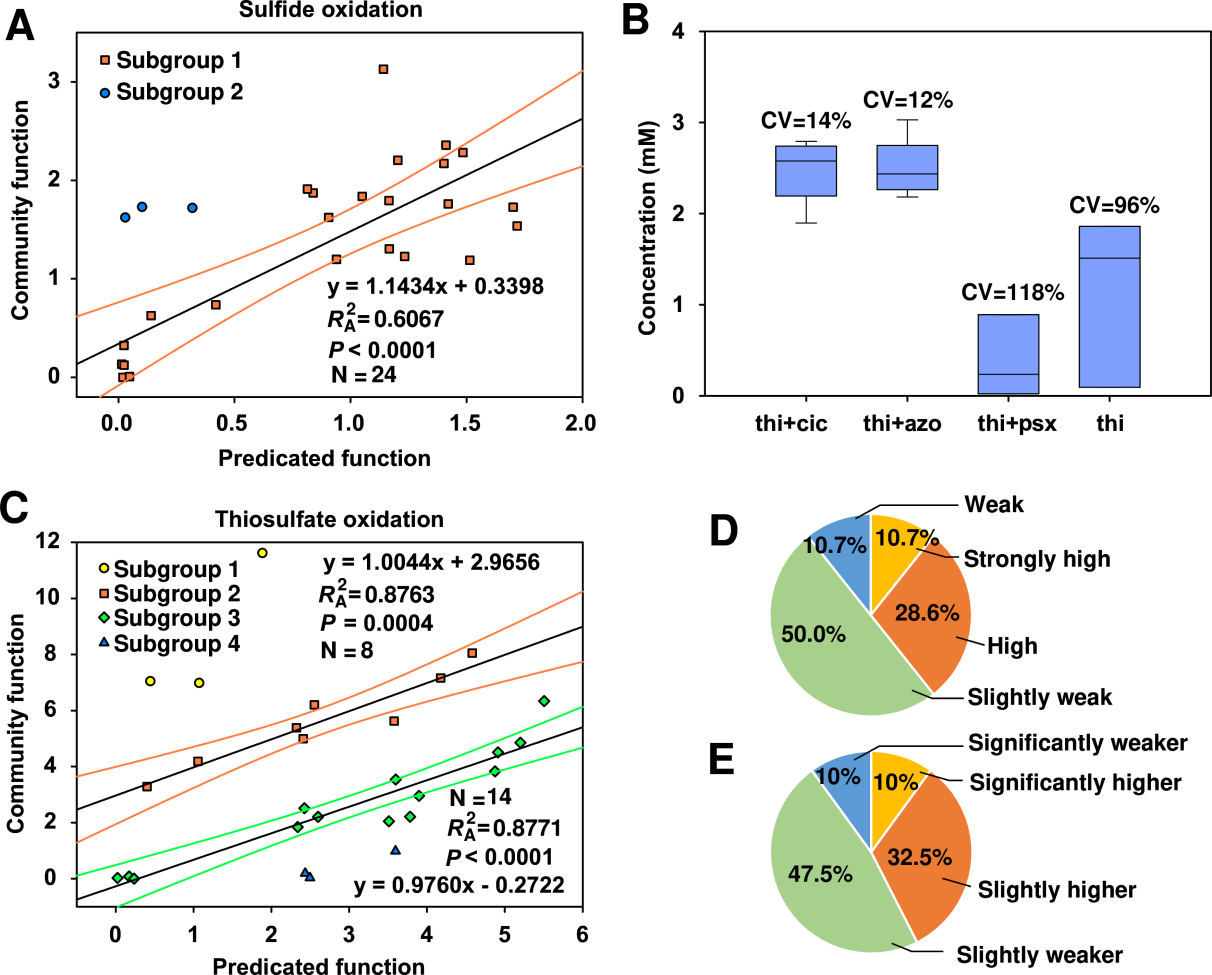
Analyses of the relationship between species’ functional traits or microbial interactions and community function. (**A**) Correlation between estimated sulfate production and measured sulfate production in the sulfide group at 95% confidence interval; (**B**) sulfate production showing the effect of microbial interactions of *Thiobacillus* with thiosulfate-oxidizing genera on community function in the sulfide group; CV, coefficient of variation showed the stability of the communities function; thi, *Thiobacillus*; cic, *Ciceribacter*; azo, *Azonexus*; psx, *Pseudoxanthomonas*; (**C**) correlation between estimated sulfate production and measured sulfate production in the thiosulfate group at 95% confidence interval; (**D**) the proportions of four subgroups in Fig. 5C showed the effect of competition on community function; (**E**) the proportions of different subgroups in the co-culturing experiment (Fig. S8) were consistent with the result of thiosulfate dilution communities (Fig. 5D).

We further investigated the linkages of the structurally differentiated communities to their function. The functional activity of a community was briefly estimated by the summation of abundance-weighted species’ functional activities. In the sulfide oxidation communities, *Thiobacillus* was the only genus with the sulfide oxidation ability under nitrate-reducing conditions, and the estimated community function (based on *Thiobacillus* abundances) was significantly (*P* < 0.001) correlated to the measured sulfate concentration (Fig. 5A). Similar results were observed in the thiosulfate oxidation group, within which the communities could be sorted into four subgroups (Fig. 5C). The observed functional activities of subgroup 2 and subgroup 3 were also significantly (*P* < 0.001) correlated to the summation of the predominant species. These results suggest that community function could be estimated by species’ function and their relative abundance.

However, there were still gaps in functional activities between the measurement and the estimation (subgroup 1 in sulfide group, Fig. 5A, subgroup 1 and subgroup 4 in thiosulfate group, Fig. 5C). The large variance between the measured and estimated functions suggested that other factors also influenced the overall function of the microbial community. Therefore, we analyzed the effect of microbial interactions on community function. Metabolic dependencies between *Thiobacillus* and those genera that oxidized thiosulfate to more sulfate (e.g., *Ciceribacter* and *Azonexus*) sustained high and stable oxidation traits of sulfide to sulfate (Fig. 5B). In contrast, metabolic dependencies between *Thiobacillus* and those thiosulfate-oxidizing genera with little sulfate production (e.g., *Pseudoxanthomonas*) slowed down the formation of sulfate (Fig. 5B). These phenomena were consistent with co-culturing experiments (Fig. 4B), indicating that metabolic cooperation could influence community function by forming different metabolic flows.

### Competition is detrimental to the stability of community function

Besides metabolic cooperation, resource competition is another type of microbial interaction shaping microbial community structure, which may affect community function. Because all isolated strains could utilize thiosulfate, they inevitably formed resource competition relationships on thiosulfate. Therefore, we performed two strains co-culturing experiments with thiosulfate as electron donors to clarify the effect of resource competition on community function. The production of sulfate was used to characterize community function. Results showed that only 10% of the co-cultures showed significantly higher activities than the mono-cultures, with 32.5% slightly higher, 47.5% slightly weaker, and 10% significantly weaker (*P* < 0.05, Fig. 5E; Fig. S8). These percentages were consistent with the percentages of the four groups in thiosulfate dilution communities (Fig. 5C and D), which indicated that competitions were mostly detrimental to the stability of community function.

## DISCUSSION

Exploring the assembly mechanisms of highly diverse and complex microbial communities and linking community structure to the ecosystem function are core issues in microbial ecology (7). Beyond the existing taxonomy-based deterministic and stochastic processes in microbial community assembly, it is believed that a deep mechanistic understanding needs the integration of analyzing species’ functional traits and interactions (16, 19 – 21). By combining high-throughput sequencing analyses with pure culturing, our study showed how species’ functional traits and interspecies’ interactions determined community structure and function. The results showed that species were different in functional traits of nitrate-mediated sulfide and thiosulfate oxidation, which determined their relative abundance in the nitrate-mediated sulfur oxidation process. Different thiosulfate-oxidizing microbes co-occurred with *Thiobacillus* by using thiosulfate secreted by *Thiobacillus* during nitrate-mediated sulfide oxidation process, which led to the different metabolic flux of sulfide oxidation. Some were conducive to maintaining the stability of sulfate production, while others changed the direction of metabolic flow with tetrathionate production. In addition, competitions among species were detrimental to the stability of the community function. These results advanced our understanding of the nitrate-mediated sulfur oxidation process and its community assembly from the perspectives of species’ functional traits and interactions. Moreover, we established quantitative links between community structure and its function which had important implications for the rational design and synthesis of microbial communities with expected functions (42).

Recently, numerous studies have been conducted to explore the mechanisms of governing community structure, and a consensus was formed that deterministic and stochastic processes jointly shaped the community structure (13, 17, 18, 55). In line with this consensus, our results showed that stochastic dispersal mainly determined the initial colonization of species, while selection dominated the later formation of local nitrate-mediated sulfur oxidation communities accompanied by stochastic drift (18, 55). The domination of selection may be due to the fact that nitrate-mediated sulfur oxidation communities are present in resource-limited habitats, and selection has been discovered to dominate community assembly in resource-limited environments (38, 39).

Analyses of species’ functional traits and interactions are considered needed for in-depth mechanistic explanations of community assembly (16, 19 – 21). By integrating pure culturing, co-culturing, and genome analysis into community assembly, our study took the lead from the perspective of both species’ functional traits and interactions in revealing the internal mechanism of community construction. The results showed that species were different in sulfide- and thiosulfate-oxidizing abilities, which determined their relative abundance in the communities. *Thiobacillus* was the only genus identified to have sulfide oxidation function in our systems, which explained why this genus dominated the nitrate-mediated sulfide oxidation process. This genus is a typical sulfur-oxidizing bacteria that harbors many genes for the oxidation of various sulfur compounds, such as sulfide, polysulfide, sulfur, thiosulfate, sulfite, etc. (28, 32, 54). Therefore, this genus is also reported predominant in many habitats involved in nitrate-mediated sulfide oxidation (28, 56 – 59).

In addition, the genera in our systems showed different functional traits in thiosulfate oxidation, which determined their relative abundances in the nitrate-mediated thiosulfate oxidation process. The *sox* gene system is a ubiquitous pathway that oxidizes thiosulfate to sulfate (60, 61). Genera harboring the *sox* gene system tend to be more likely to dominate the thiosulfate oxidation process. The *tsd*A or *dox*A are genes encoding enzymes that oxidize thiosulfate to tetrathionate but with lower productivity than the *sox* gene system (62 – 64). Therefore, genera containing these genes for thiosulfate oxidation dominated only in higher dilution where genera harboring the *sox* gene system were absent. The *tst* is another gene for the oxidation of thiosulfate to sulfite relying on cyanide (65, 66). In the condition that cyanide is lacking, the oxidation of thiosulfate by this gene would be restricted. Therefore, genera contained solely the *tst* gene for thiosulfate oxidation occurred only when genera harboring the *sox* or *tsd*A genes were absent. The above functional differences reflected the selection process of niche partitioning and niche competition among species (67 – 70). Under the circumstance of niche competition, trait equalizing is a mechanism leading to species coexistence (69, 70). Therefore, species with similar thiosulfate-oxidizing abilities may coexist with close abundances in the communities (Fig. 1C).

In addition to species’ functional differences, our results showed that metabolic interaction was a key process leading to microbial species coexistence, which also affected the community structure. The sulfide oxidation process of *Thiobacillus* might secrete thiosulfate, which supports the growth of other thiosulfate oxidation genera (54, 56, 57). Generally, metabolic interactions are widespread among microbial species (22, 24, 40, 71). For instance, metabolite secretion is a process by which species modify their environment, which would create new niches for other species (72). Previous studies have shown that thiosulfate was indeed generated in the process of sulfide oxidation by *Thiobacillus* (54, 56, 57). The generation of thiosulfate could be a reason why many microorganisms (e.g., *Thermomonas*, *Rhizobium*, and *Ochrobactrum*) coexisted with sulfide-oxidizing bacteria in the community although they do not have sulfide oxidation functions (32, 56, 58, 59). Notably, species that relied on metabolites to survive often had relatively low abundances. We consider that this is possibly limited by the concentration of metabolites.

The growth and reproduction of microorganisms not only shape the community structure, but their metabolic activities also affect the substrate circulation of the system (73, 74). Clarifying the relationship between community structure and function is important for understanding mechanisms governing ecological processes and therefore taking effective measures to regulate ecosystem function (2, 3, 14, 74). Our results showed that both the sulfide and thiosulfate oxidation traits of the systems were in linear relation with the summation of abundance-weight functions of its key species, indicating that the system function can be quantitatively estimated by the function and abundance of the species. This is the basis for describing community function based on species relative abundance (27).

However, the function of the community is not completely equal to the simple summation of species functions. Microbial interactions also had an important impact on the community function. On the one hand, the metabolic dependencies of those genera containing *sox* gene system on *Thiobacillus* were beneficial to the stability of sulfate production. This was consistent with many previous reports that metabolic cooperation can improve the stability and robustness of community function (24, 75, 76). On the other hand, less sulfate generated in communities of *Thiobacillus* co-occurred with genera that contain the *tsd*A gene, transforming thiosulfate to tetrathionate (e.g., *Achromobacter*). Therefore, microbial interactions may change the direction of the metabolic flow due to the formation of different pathways (75). These metabolic conjugation processes may explain the bio-formation of different sulfur compounds in natural habitats (77).

Besides metabolic cooperation, there are many other types of microbial interactions, such as competition, antagonism, parasitism, and predation, whose effects on community function were rarely explored (78). For instance, resource competition among species could result in mutual exclusion and might lead to the turbulence of community structure and function (79). Indeed, our results showed that species in thiosulfate oxidation communities mainly competed for thiosulfate, and these communities characterized by competition were highly divergent in both structure and function. Therefore, competitions were mostly detrimental to the stability of community function.

Taken together, our results revealed that species’ functional traits and interactions were the intrinsic factors determining community structure and function. To our knowledge, our study took the lead from the perspective of both species’ functional traits and interactions in providing mechanistic explanations for the community assembly. Moreover, our study established quantitative links between community structures with their function, indicating that the community function could be regulated by microbial interactions. For instance, by avoiding competition or building metabolic cooperation between species, microbial interactions may improve the function and stability of the community or change the flow direction of metabolism (42). Therefore, our study also has important implications for designing and constructing microbiomes with expected functions.

Notably, our study was performed in simplified laboratory systems and mainly investigated the functions and interactions of predominant species in the microbial community. Natural microbial communities are highly complex and variable, and contain many rare species (80). It is believed that rare species play important roles in ecosystem functioning, contributing to functional diversity and stability via functional redundancy (81, 82). The higher and more stable functional activities observed in less diluted samples (Fig. S2) may account for the presence of more rare species. Further studies need to take rare species into account, combined with the analyses of species’ functional traits and interactions, to fully and deeply uncover the mechanisms governing microbial community assembly and to link microbial community structure to its function.

## Data Availability

The raw 16S rRNA sequencing data have been deposited in National Omics Data Encyclopedia (NODE) with accession numbers from OED831055 to OED831188 (https://www.biosino.org/node/run/detail/OER420660) and National Center for Biotechnology Information with accession numbers from SRR24564454 to SRR24564520 (https://www.ncbi.nlm.nih.gov/Traces/study/?acc=SRP437722&o=acc_s%3Aa).

## SUPPLEMENTAL MATERIAL

Text S1 mbio.01567-23-s0001.docx Supplemental text.

Fig. S1 mbio.01567-23-s0002.docx Schematic for community self-assembly and functional analyses.

Fig. S2 mbio.01567-23-s0003.docx Variation of community function along a gradient of dilution.

Fig. S3 mbio.01567-23-s0004.docx Shannon indices showed the simplification of dilution culturing communities.

Fig. S4 mbio.01567-23-s0005.docx Family composition of diluted culturing communities for sulfide and thiosulfate groups.

Fig. S5 mbio.01567-23-s0006.docx Order composition of diluted culturing communities for sulfide and thiosulfate groups.

Fig. S6 mbio.01567-23-s0007.docx Generation of tetrathionate by isolated strains from nitrate-mediated thiosulfate oxidation.

Fig. S7 mbio.01567-23-s0008.docx Verifying thiosulfate as one of the intermediates produced by *Thiobacillus* which supported the growth of other thiosulfate oxidizers.

Fig. S8 mbio.01567-23-s0009.docx The comparison of sulfate productions between two strains co-culturing in the nitrate-mediated thiosulfate oxidation system and mono-culturing after 4 days of incubation.

Table S1 mbio.01567-23-s0010.docx Supplemental tables.

ASM does not own the copyrights to Supplemental Material that may be linked to, or accessed through, an article. The authors have granted ASM a non-exclusive, world-wide license to publish the Supplemental Material files. Please contact the corresponding author directly for reuse.
