# Supplementary material for: Species’ functional traits and interactions drive nitrate-mediated sulfur-oxidizing community structure and functioning
Source: mBio. 2023 Sep 13;14(5):e01567-23. doi: 10.1128/mbio.01567-23 (PMC10653917; doi:10.1128/mbio.01567-23)
Supplement: Text S1 — Supplemental text. [file mbio.01567-23-s0001.docx]

**Supplemental text**

**Text S1A. Measurement of potential nitrate-mediated sulfur oxidation activities for the sedimentary species pool**

The sediment sample was tested for potential nitrate-mediated sulfide oxidation activities and potential nitrate-mediated thiosulfate oxidation activities, respectively using medium NS (containing 2 g/l KNO_3_, 1 g/l Na_2_S⸱9H_2_O, 1 g/l NaHCO_3_, 0.5 g/l FeCl_2_, 2 g/l KH_2_PO_4_ and 0.1 g/l MgCl_2_) and medium NT (containing 2 g/l KNO_3_, 5 g/l Na_2_S_2_O_3_⸱5H_2_O, 1 g/l NaHCO_3_, 0.1 g/l FeCl_2_, 2 g/l KH_2_PO_4_ and 0.1 g/l MgCl_2_). 0.1 g of sediment was added to a 10 ml medium in a 20 ml headspace bottle. After nitrogen blowing, samples were placed in an anaerobic chamber and stationary cultured at 30 ℃. The potential nitrate-mediated sulfur oxidation activities was calculated by the consumption of nitrate and the production of sulfate after two days of incubation.

**Text S1B. Measurement of physicochemical parameters**

The concentration of nitrate, nitrite, thiosulfate and sulfate were measured using a ThermoFisher Scientific Ion Chromatography ICS-1100 equipped with an AS-25 column. The sediment pore water was diluted by 5-folds and filtered using 0.22 μm membrane filters before testing. The artificial medium was diluted by 50-folds and filtered using 0.22 μm membrane filters before testing. The AVS (acid-volatile sulfide) of sediment was measured according to the method previously described (1).

**Text S1C. Measurement of nitrate-mediated sulfur oxidation traits for isolated strains**

Cells of isolated strains were collected after culturing in agar plates with the NT medium and inoculated (biomass ~10^7^ cells/ml) into a fresh 10 ml medium containing 2 g/l KNO_3_, 1 g/l NaHCO_3_, 0.5 g/l FeCl_2_, 2 g/l KH_2_PO_4_, and 0.1 g/l MgCl_2_. Sulfide, sulfur and thiosulfate (1 g/l Na_2_S⸱9H_2_O, 0.5 g/L S^0^ and 5 g/l Na_2_S_2_O_3_⸱5H_2_O, respectively) were added to the medium separately for testing nitrate-mediated sulfide, sulfur and thiosulfate oxidation traits of the isolated strains. The nitrate consumption, nitrite production, and sulfate production rates were measured after five days of incubation at 30 ℃.

**Text S1D. Q-PCR for measuring the growth of strains in co-culturing**

The amplified nearly full-length 16S rRNA gene of each strain was separated and extracted from the gal to evaluate the qPCR amplification efficiency and establish the standard curve. The qPCR amplification was performed on a CFX Connect^TM^ Real-Time System (BIO-RAD) by pre-heating at 95 ℃ for 3 min, followed by 45-three segment cycles of denaturation at 94 ℃ for 15 sec, annealing at 50 ℃ for 5 sec (Table S1B), extension at 72 ℃ for 30 sec and a final extension at 72 ℃ for 5 min. DNA templates for qPCR were extracted and purified form the same volume of culture and identical protocol as described previously (2).

**Text S1E. Additional experiment conducted to verify thiosulfate as one of the intermediates produced by *Thiobacillus* that supports the growth of other thiosulfate oxidizers**

The strain of *Thiobacillus* was cultured in 250 ml medium NS for two days at 30 ℃. The resulting culture solution was filtered by 0.22 μm membranes to remove cells. Half of the filtered culture solution was treated by iodine (I_2_), with a final concentration of 10 mg/L, to remove thiosulfate by oxidizing it to tetrathionate. Then the iodine-treated and untreated solution was used for culturing *Ciceribacter* and *Pseudoxanthomonas*, respectively, in 20 ml headspace bottles containing 10 ml liquid. After being purged by N_2_ to remove oxygen, all samples were placed in an anaerobic chamber and stationary cultured at 30 ℃ for two days. The concentrations of sulfate, thiosulfate and tetrathionate were measured using a ThermoFisher Scientific Ion Chromatography ICS-1100 equipped with an AS-11 column. Bacterial growth was assessed by monitoring the amplification of the partial 16S rRNA gene using strain-specific primers (Table S1B) via qPCR. Additionally, glucose was added into both the iodine-treated and untreated solutions, with a final concentration of 3 g/L, and used for culturing *Ciceribacter* and *Pseudoxanthomonas* separately in tubes to test the effect of iodine on the growth, which was measured by a spectrophotometer at a wavelength of 600 nm (OD_600_) after 24 hours incubation.

**References**

1. Allen HE, Fu GM, Deng BL. 1993. Analysis of acid-volatile sulfide (AVS) and simultaneously extracted metals (SEM) for the estimation of potential toxicity in aquatic sediments. Environ Toxicol Chem 12:1441-1453.
2. Zhou J, Bruns MA, Tiedje JM. 1996. DNA recovery from soils of diverse composition. Appl Environ Microbiol 62:316-22.
