# Supplementary figures and images for: Species’ functional traits and interactions drive nitrate-mediated sulfur-oxidizing community structure and functioning
Source: mBio. 2023 Sep 13;14(5):e01567-23. doi: 10.1128/mbio.01567-23 (PMC10653917; doi:10.1128/mbio.01567-23)

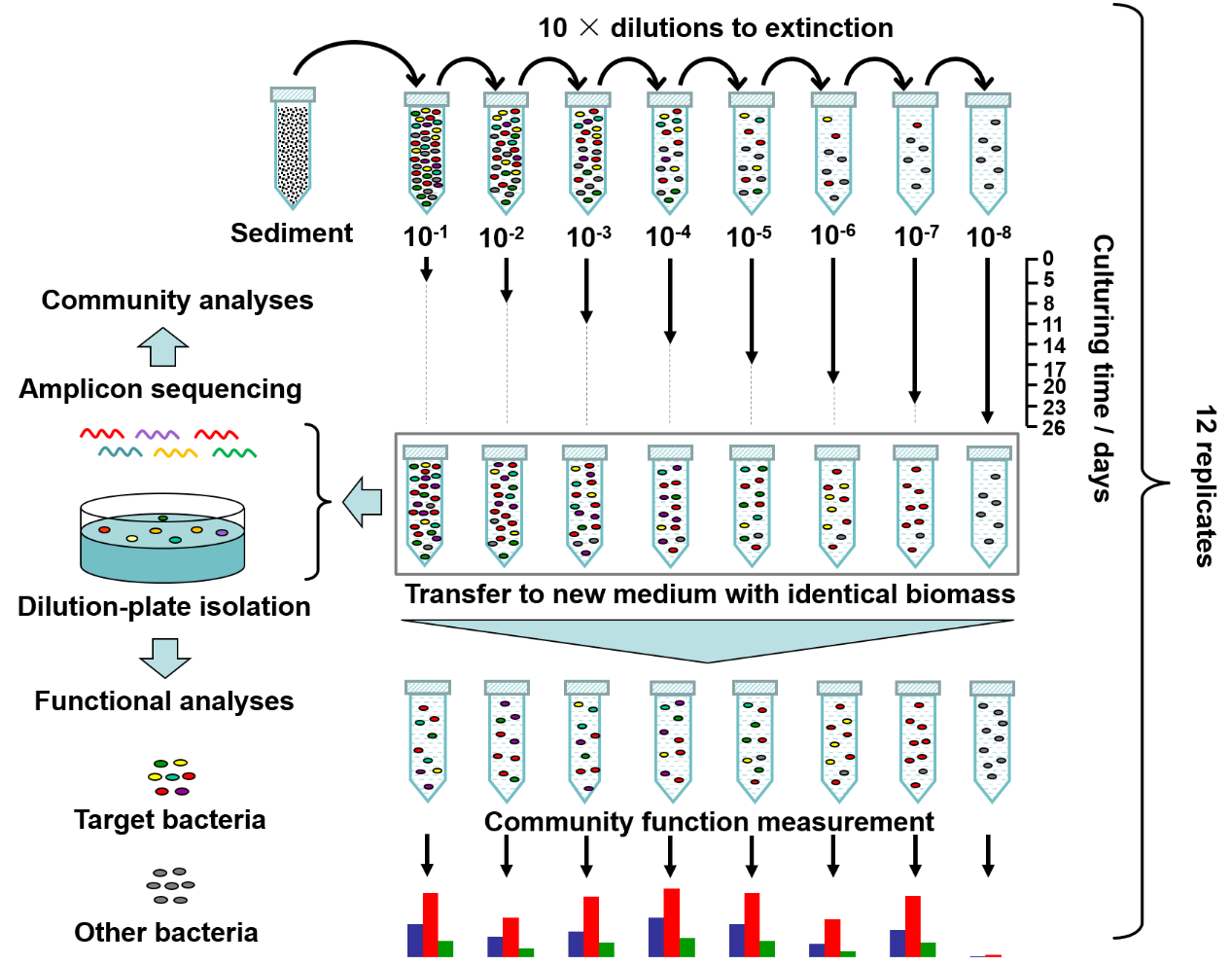


**Fig. S1.** Schematic for community self-assembly and functional analyses.

Supplement: Fig. S1 — Schematic for community self-assembly and functional analyses. [file mbio.01567-23-s0002.docx]
