# Supplementary material for: Species’ functional traits and interactions drive nitrate-mediated sulfur-oxidizing community structure and functioning
Source: mBio. 2023 Sep 13;14(5):e01567-23. doi: 10.1128/mbio.01567-23 (PMC10653917; doi:10.1128/mbio.01567-23)
Supplement: Fig. S2 — Variation of community function along a gradient of dilution. [file mbio.01567-23-s0003.docx]

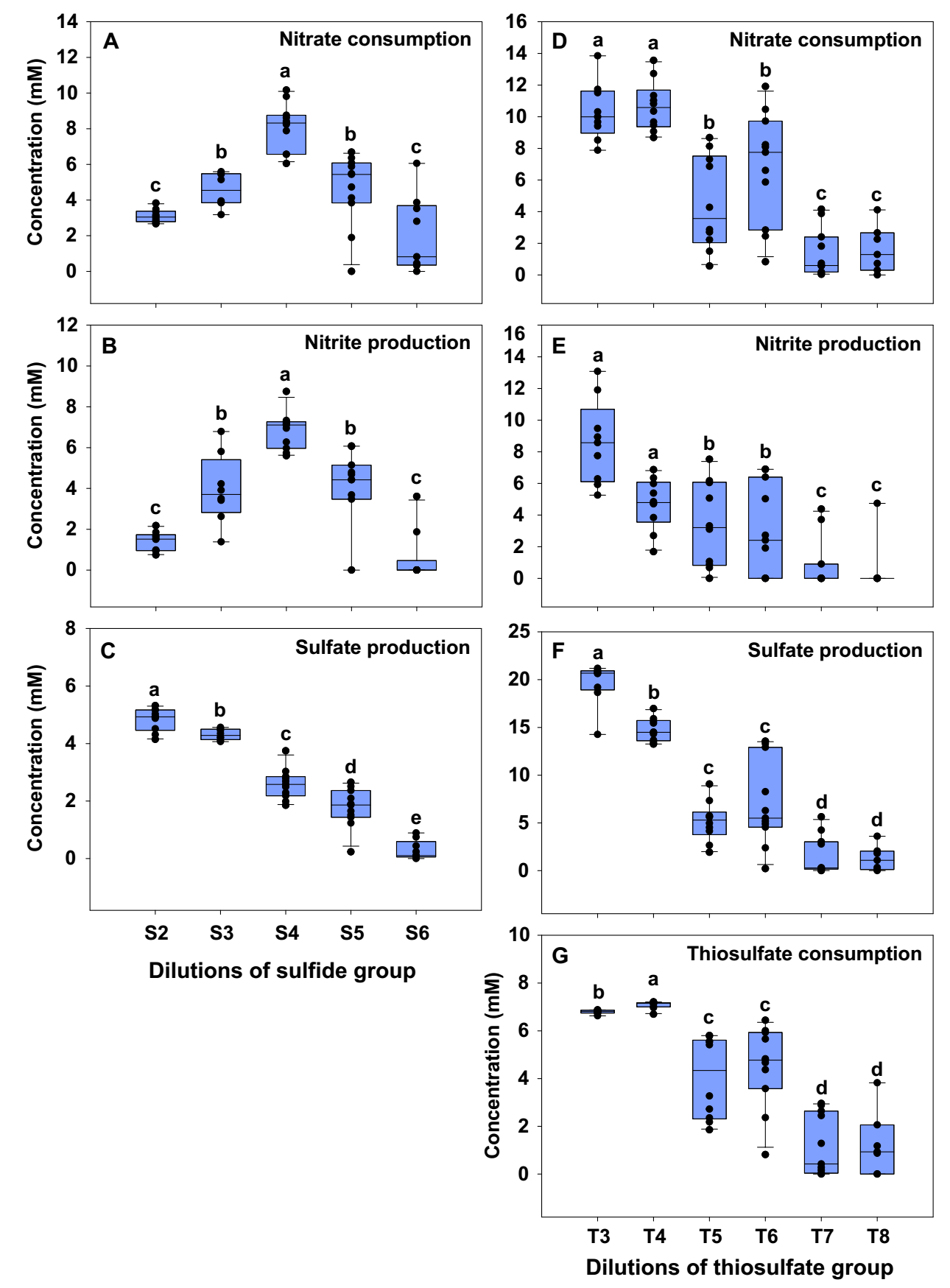


**Fig. S2.** Variation of community function along a gradient of dilution. A-C showed nitrate consumption, nitrite production and sulfate production of dilution communities of the sulfide group after 5 days of incubation, respectively. D-G respectively showed nitrate consumption, nitrite production, thiosulfate consumption and sulfate production of dilution communities of the thiosulfate group after 5 days of incubation, respectively. S2-S6 indicated 10^-2^-10^-6^ dilutions of the sulfide group, respectively; T3-T8 indicated 10^-3^-10^-8^ dilutions of the thiosulfate group, respectively. a, b, c, d, e represented significant differences between groups with *p* < 0.05 by one way ANOVA.
