# Supplementary material for: Species’ functional traits and interactions drive nitrate-mediated sulfur-oxidizing community structure and functioning
Source: mBio. 2023 Sep 13;14(5):e01567-23. doi: 10.1128/mbio.01567-23 (PMC10653917; doi:10.1128/mbio.01567-23)
Supplement: Fig. S3 — Shannon indices showed the simplification of dilution culturing communities. [file mbio.01567-23-s0004.docx]

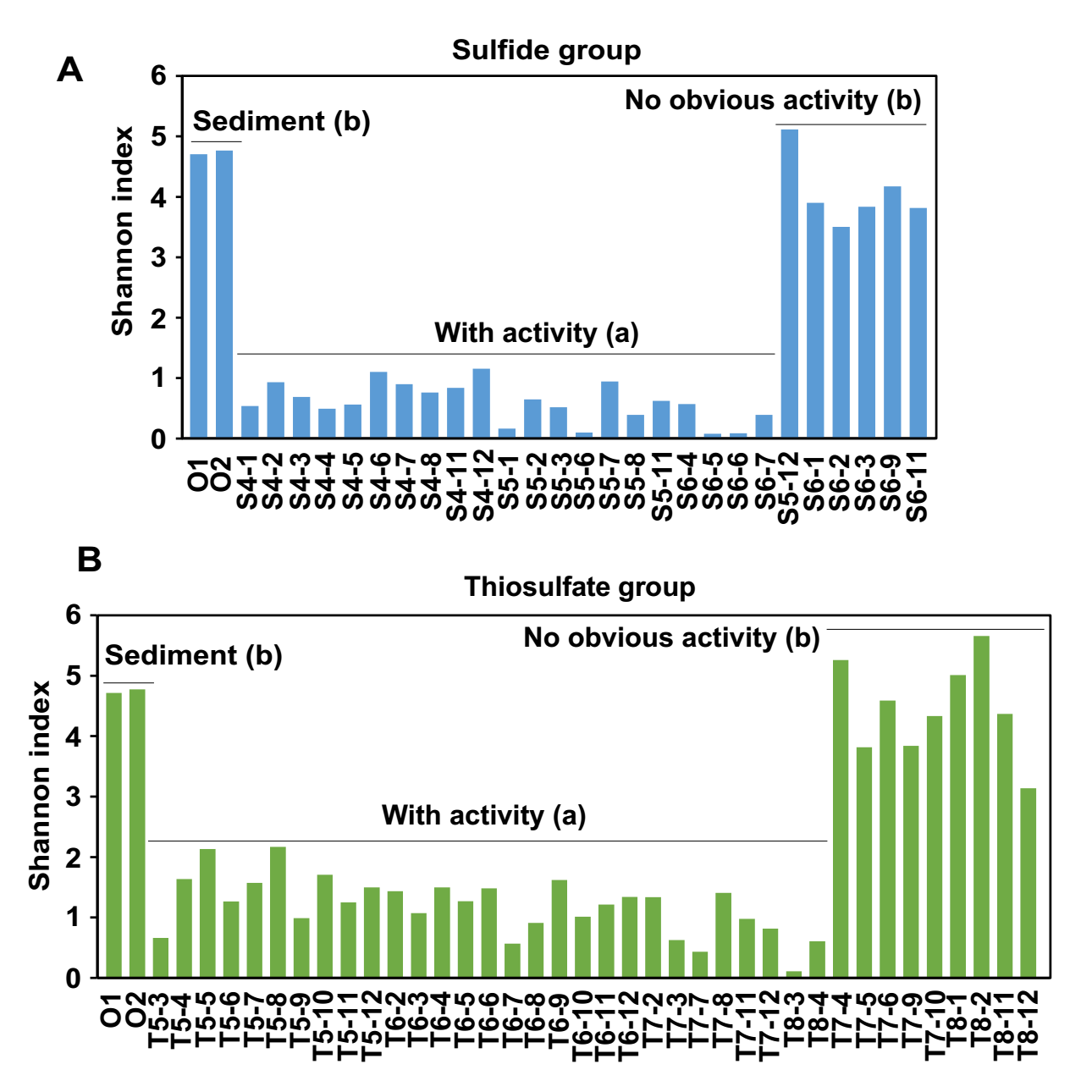


**Fig. S3.** Shannon indices showed the simplification of dilution culturing communities with obvious nitrate-mediated sulfide and thiosulfate oxidation activities for communities of sulfide- (A) and thiosulfate-oxidation communities (B). a, b in parentheses represented significant differences between groups with *p* < 0.001 by one way ANOVA.
