# Supplementary material for: Species’ functional traits and interactions drive nitrate-mediated sulfur-oxidizing community structure and functioning
Source: mBio. 2023 Sep 13;14(5):e01567-23. doi: 10.1128/mbio.01567-23 (PMC10653917; doi:10.1128/mbio.01567-23)
Supplement: Fig. S6 — Generation of tetrathionate by isolated strains from nitrate-mediated thiosulfate oxidation. [file mbio.01567-23-s0007.docx]

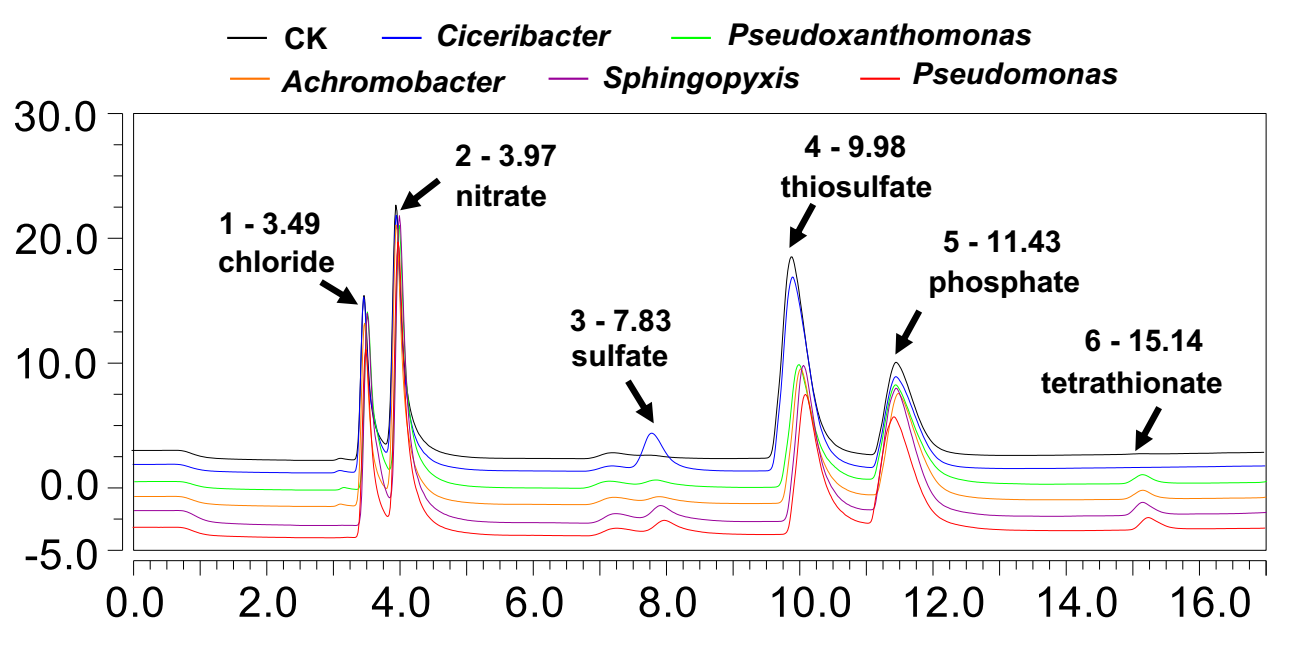


**Fig. S6.** Generation of tetrathionate by isolated strains from nitrate-mediated thiosulfate oxidation.
