# Supplementary material for: Species’ functional traits and interactions drive nitrate-mediated sulfur-oxidizing community structure and functioning
Source: mBio. 2023 Sep 13;14(5):e01567-23. doi: 10.1128/mbio.01567-23 (PMC10653917; doi:10.1128/mbio.01567-23)
Supplement: Fig. S7 — Verifying thiosulfate as one of the intermediates produced by Thiobacillus which supported the growth of other thiosulfate oxidizers. [file mbio.01567-23-s0008.docx]

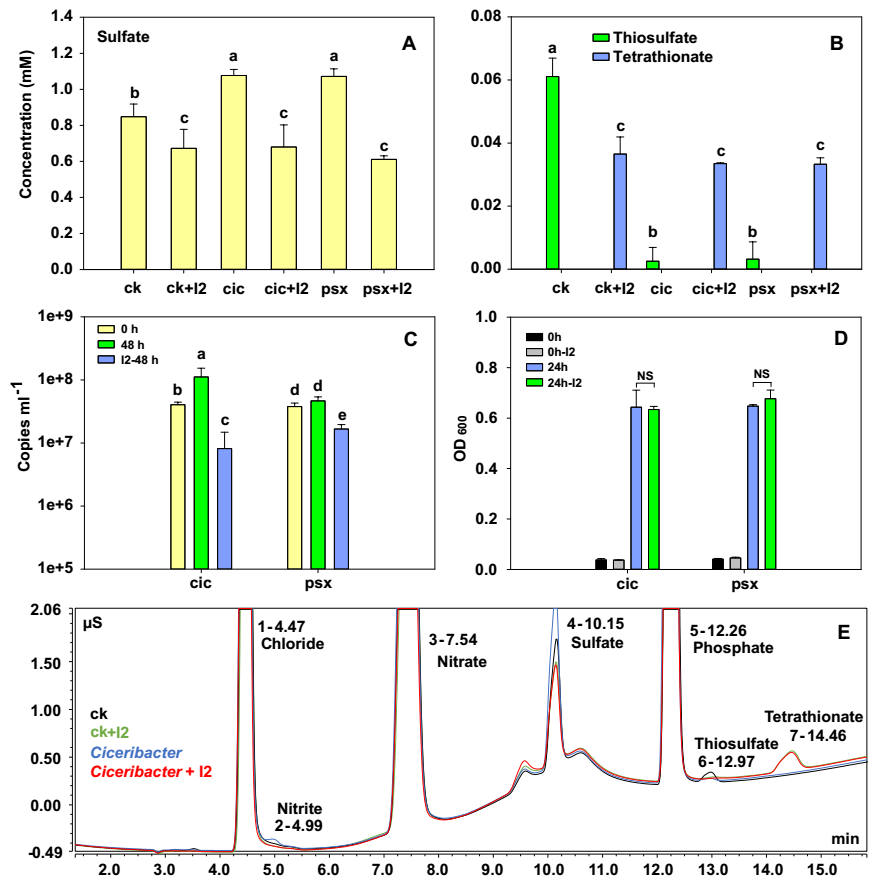


**Fig. S7.** Verifying thiosulfate as one of the intermediates produced by *Thiobacillus* which supported the growth of other thiosulfate oxidizers. A, the removal of thiosulfate from the medium led to less sulfate production; B, thiosulfate was oxidized by thiosulfate oxidizers and by iodine, respectively; C, the copy numbers of thiosulfate oxidizers decreased when thiosulfate was removed; D, adding iodide in the medium had no inhibition effect on the growth of *Ciceribacter* and *Pseudoxanthomonas* using glucose; E, ion chromatogram showed changes of anions in the medium for different treatments; a, b, c, d, e, represented significant different groups with *p* < 0.05 by one way ANOVA; NS, no significant difference by independent samples *t*-test; ck, abiotic control; I2, medium treated with 10 mg/L iodide to remove thiosulfate; cic, *Ciceribacter*; psx, *Pseudoxanthomonas*.
