# Supplementary material for: Species’ functional traits and interactions drive nitrate-mediated sulfur-oxidizing community structure and functioning
Source: mBio. 2023 Sep 13;14(5):e01567-23. doi: 10.1128/mbio.01567-23 (PMC10653917; doi:10.1128/mbio.01567-23)
Supplement: Fig. S8 — The comparison of sulfate productions between two strains co-culturing in the nitrate-mediated thiosulfate oxidation system and mono-culturing after 4 days of incubation. [file mbio.01567-23-s0009.docx]

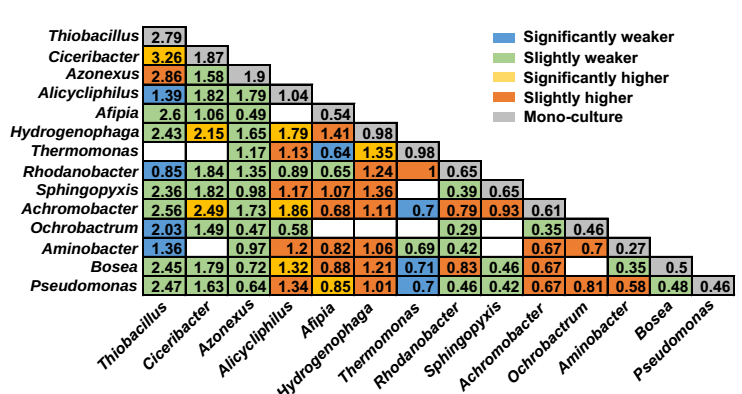


**Fig. S8.** The comparison of sulfate productions between two strains co-culturing in the nitrate-mediated thiosulfate oxidation system and mono-culturing after 4 days of incubation. Those co-cultures that produced significantly more sulfate than both of their mono-cultures were defined as “Significantly higher”. Those co-cultures that produced more sulfate than both of their mono-cultures were defined as “Slightly higher”. Those co-cultures that produced less sulfate than any one of their mono-cultures were defined as “Slightly weaker”. Those co-cultures that produced significantly less sulfate than any one of their mono-cultures were defined as “Significantly weaker”. Significance was tested between co-culture and corresponding mono-culture at *p* < 0.05 by independent samples *t*-test.
