# Supplementary material for: Species’ functional traits and interactions drive nitrate-mediated sulfur-oxidizing community structure and functioning
Source: mBio. 2023 Sep 13;14(5):e01567-23. doi: 10.1128/mbio.01567-23 (PMC10653917; doi:10.1128/mbio.01567-23)
Supplement: Table S1 — Supplemental tables. [file mbio.01567-23-s0010.docx]

**Table S1A.** Physicochemical parameters of the sedimentary species pool.

| Site | RG1 | RG2 | RG3 | RG4 | Average | Stdev |
| --- | --- | --- | --- | --- | --- | --- |
| AVS(mg/g-dry sediment) | 1.04 | 1.66 | 2.91 | 1.29 | 1.73 | 0.83 |
| Nitrate(mmol/L) | 0.08 | 0.08 | 0.07 | 0.11 | 0.08 | 0.02 |
| Sulfate(mmol/L) | 3.64 | 3.83 | 4.34 | 2.35 | 3.54 | 0.85 |
| S-PRN (mmol/L) | 8.06 | 3.31 | 8.03 | 4.14 | 5.89 | 2.52 |
| S-PPS (mmol/L) | 3.12 | 2.80 | 3.12 | 2.41 | 2.86 | 0.34 |
| T-PRN (mmol/L) | 15.80 | 14.76 | 15.06 | 9.37 | 13.75 | 2.95 |
| T-PPS (mmol/L) | 10.00 | 9.60 | 6.63 | 9.33 | 8.89 | 1.53 |

S-PRN, potential removal rate of nitrate for nitrate-mediated sulfide oxidation; S-PPS, the potential production rate of sulfate for nitrate-mediated sulfide oxidation; T-PRN, the potential removal rate of nitrate for nitrate-mediated thiosulfate oxidation; T-PPS, the potential production rate of sulfate for nitrate-mediated thiosulfate oxidation.

**Table S1B.** Quantitative PCR primer pair and respective annealing temperature of each genus designed by 16S rRNA gene sequences of respective strain.

| Strain | Genus | Primer name | Primer sequence | Annealing temperature |
| --- | --- | --- | --- | --- |
| **S5643** | *Thiobacillus* | thi8f | GGGAGTGAAATCCCTTAG | 50 |
|  |  | thi9r | CTCTGCAGGGTTCTGGA |  |
| **S431** | *Ciceribacter* | cic8f | GTCGGGCAGTTGACTGT | 50 |
|  |  | cic9r | CCTAACTGAAGGACAATGT |  |
| **S544** | *Azonexus* | azo8f | GGTGGGTAAAACCATTTAG | 50 |
|  |  | azo9r | TCTCTTCAGGATTCTGAAC |  |
| **T572** | *Alicycliphilus* | ali8f | TGTTGGGGATTAATTTTCTC | 50 |
|  |  | ali9r | CTCTTCGGGATTCCATAC |  |
| **T677** | *Afipia* | afi9f | GGTCGCAGAGATGTGAC | 50 |
|  |  | afi12r | GGTCGCCCCTTTGCAT |  |
| **S685** | *Pseudoxanthomonas* | psx4f | GTTGGGAAAGAAATCCTATC | 50 |
|  |  | psx6r | CTACCACACTCTAGTGAC |  |
| **T568** | *Thermomonas* | the4f | TCCGGAAAGAAAAGCATTC | 50 |
|  |  | the6r | TACACCAGGAATTCCACTA |  |
| **T83** | *Sphingopyxis* | spi8f | GGGCTCATAGAGCTTGG | 50 |
|  |  | spi9r | ATCTCTGGTAACCGCGAT |  |
| **T763** | *Pseudomonas* | pse4f | GTAAGTTAATACCTTGCTGT | 50 |
|  |  | pse6r | AGCTCGCCAGTTTTGGAT |  |
| **T5712** | *Achromobacter* | ach4f | GTTAATACCCCGTGAAACT | 50 |
|  |  | ach6r | CTCTAGCCCGGTAGTTAA |  |

**Table S1C.** Coefficients of variation for functional traits of different dilution communities.

| Group | Dilution | Nitrate | Nitrite | Sulfate | Thiosulfate |
| --- | --- | --- | --- | --- | --- |
| Sulfide group | S2 | 11.81 | 33.59 | 8.05 | ND |
|  | S3 | 20.85 | 43.17 | 4.32 | ND |
|  | S4 | 16.05 | 13.22 | 20.56 | ND |
|  | S5 | 44.67 | 53.70 | 38.64 | ND |
|  | S6 | 104.95 | 223.64 | 113.17 | ND |
| Thiosulfate group | T3 | 17.60 | 30.92 | 11.21 | 1.20 |
|  | T4 | 14.72 | 34.24 | 8.17 | 2.25 |
|  | T5 | 66.00 | 79.62 | 40.39 | 42.02 |
|  | T6 | 51.76 | 98.57 | 64.40 | 37.68 |
|  | T7 | 108.82 | 198.74 | 131.54 | 107.19 |
|  | T8 | 90.68 | 264.58 | 101.40 | 105.33 |

ND, No data.
